# The Role of Information in Evolutionary Biology

**DOI:** 10.1007/s10441-023-09468-4

**Published:** 2023-05-15

**Authors:** Thomas E. Dickins

**Affiliations:** 1 Faculty of Science & Technology, Middlesex University, London, U.K.; 2 Centre for Philosophy of Natural and Social Science, London School of Economics, London, U.K.

**Keywords:** Gene centrism, DNA, Information, Data, Developmental program, Reaction norms, Evo-devo

## Abstract

The Modern Synthesis has received criticism for its purported gene-centrism. That criticism relies on a concept of the gene as a unit of instructional information. In this paper I discuss information concepts and endorse one, developed from Floridi, that sees information as a functional relationship between data and context. I use this concept to inspect developmental criticisms of the Modern Synthesis and argue that the instructional gene arose as an idealization practice when evolutionary biologists made comment on development. However, a closer inspection of key claims shows that at least some associated with the Modern Synthesis were in fact adopting the data led definition I favour and made clear arguments for the role of developmental processes beyond genetic input. There was no instructional gene.

## Introduction

Calls for an extension of the Modern Synthesis^1^ in evolutionary biology arise from two broad approaches. One is the consideration of recent findings in biology that might be interpreted as challenges to understanding gained under the Modern Synthesis. The other is more philosophically grounded in the explanatory architecture of the Modern Synthesis. Within this second approach a significant amount of work has sought to criticize what is often referred to as *gene-centrism*. This refers to specific theoretical commitments associated with the genes’ eye view, best articulated by Dawkins and Williams in the second half of the 20th century (Dawkins 1976; Williams 1996; Ågren 2021). The principal criticisms of this perspective are that (i) genes have been given a comprehensive instructional role at the expense of other developmental processes (Pigliucci 2007; Newman 2010; Sultan 2019), and (ii) genetic variation might not be the only source of variation for evolutionary process (Gerhart and Kirschner 2007; Danchin et al. 2011; Kirschner 2013). Both points are often brought into contact with recent findings in biology to clarify the claims being made. This package of criticisms has been referred to as the developmental challenge to the Modern Synthesis (Love 2017). This article will focus upon the first argument.

Those advocating for an extension to the Modern Synthesis, and those who have supported the synthetic view, often deploy concepts of information (e.g., Maynard Smith 2000; Godfrey-Smith 2007a; Danchin 2013; Griffiths 2017). Despite this, the concept of information has not been formally incorporated into biology, but rather adopted as an analogy or metaphor (Kay 2000; Avery 2012). By inspecting Maynard Smith’s (2000) key paper on the concept of information in biology I will show where the view of the gene as an instructional unit finds initial support. However, a closer reading of that paper shows that Maynard Smith is not advocating a fully instructional view. I will make this case and argue that a more subtle view of information was implicit in the Modern Synthesis. Maynard Smith’s analysis of information relies on modelling via analogy but also idealization, a topic that has been central to recent work on the broader topic of scientific understanding (Elgin 2007; de Regt 2017; Levy 2018; Potochnik 2020; Frigg 2022). This leads me to argue that those scholars launching a developmental challenge to the paradigm of the Modern Synthesis have a different explanatory agenda, one not in conflict with that of the Synthesis.

The paper will be organized as follows: in Sect. 2 I introduced the concept of gene centrism and then the criticism from development. Section 3 focuses upon Maynard Smith’s analysis of the information concept in biology, relating it to its origins with Dretske’s ideas and showing how it goes beyond them. In Sect. 4 I apply this view of information to the developmental challenges raised in Sect. 2, and then in Sect. 5 I draw concluding comments.

## Gene-centrism: Instruction Versus Development

In this section I will briefly discuss gene-centric models in evolution (2.1) and the claim that genes have been given a comprehensive instructional role at the expense of developmental processes (2.2).

### Optimality Models and Developmental Programs

The latter stages of the Modern Synthesis saw the introduction of what has been termed the gene’s eye view of evolution, a view that was further advanced in the years immediately following the period of the synthesis (Ågren 2021; (Dickins 2021a)^2^. A major aspect of this view was the distinction between replicators and vehicles that Dawkins derived from theoretical advances in the understanding of social behaviour (Dawkins 1976)^3^. Genes, as replicators, have the properties of copying fidelity, fecundity, and longevity. These properties enable them to persist across generations and form long lineages of identical copies. Bodies act as vehicles for genetic replicators and facilitate replication via reproduction. But vehicles themselves are short-lived and their reproduction is not replication because identical vehicles are not produced.

Genetic replicators can be modelled as strategic agents, contributing to vehicular traits that enable their replication. This insight is related to a modelling approach known as the phenotypic gambit in which organisms are treated as haploid (Grafen 1984). To take the gambit, a particular phenotypic trait is assumed to be minimally caused, in the sense of underpinned, by one gene that might occur in different forms (alleles). Thus, alternative forms of the phenotype are due to different alleles. The gambit is not a serious commitment to single-gene molecular biology, but instead is a form of idealization for improving understanding, and as such it relies on a simplifying untruth about the complexity of genetic causes (Elgin 2007; Potochnik 2008, 2013, 2020). With this simplification in place optimality models can be constructed that track the relative success of particular gene-trait complexes and can help to understand the evolutionary dynamics that will lead to some traits becoming evolutionarily stable strategies (Maynard Smith 1982).

An early example of this modelling strategy can be found in a theoretical paper on the evolution of siblicide in Kittiwakes (*Rissa tridactyla*), which is thought to be a method of brood reduction (O’Connor 1978; Dickins and Clark 1987). Dickins and Clark hypothesized two types of Kittiwake chick: an ejector, *E*, that will always push its sibling out of the nest to its death, and a huddler, *H*, that will hunker down and attempt to avoid conflict. The conceit of such models is to consider how one allele may come to dominate over another in the population, or whether they can co-exist in equilibrium. A typical model may start by assuming a population entirely composed of huddlers and ask what would happen should a mutant *E* allele arise in the population? How might that spread across generations? This relies upon a fitness coefficient being attached to each allele, or phenotype.

Dickins and Clark attached a probability of survival^4^ to each kind of chick and created a pay-off matrix showing that *E-E* interactions carry a 0.5 survival probability for each, as only one can win; *E-H* a probability of 1.0 for the ejector and 0 for the huddler; and a *H-H* a probability of 1.0 as neither will attack. The researchers made explicit reference to other simplifying assumptions, including that of asexual reproduction. Given these parameters, Dickins and Clark were then able to calculate the average payoffs to ejectors and huddlers over time, and the impact of different starting ratios of each type for population composition across generations, which in turn showed how each strategy could come to dominate. In this way an evolutionary model was produced that enabled researchers to make better sense of the behaviours they were seeing in real populations of Kittiwakes. In short, it made it more likely that observations of siblicide were observations of adapted behaviour rather than anomalies or accidents.

As is usual in evolutionary scenarios, selection is interacting with the phenotype in the Kittiwake model. But, in keeping with the gene’s eye view it is acting to sift gene frequencies in the gene pool. Those gene frequencies are artificial due to the single allele assumption inherent in the phenotypic gambit, as well as the assumption of asexuality. Maynard Smith (1982: 20–22) was clear that filling in the true details obscured by these assumptions would be onerous but would have no qualitative impact upon the outcome of the model. This is done for simplicity to capture evolutionary dynamics and to ask whether a particular phenotype carries a positive fitness advantage.

It is within this gene’s eye framework that critics have noted another assumption. The idealization of a complex polygenic underpinning to a single allele appears to bring with it the companion assumption that only genes cause the phenotype. Mapping the phenotypic trait to one or to many genes places developmental control firmly at the genetic level. This view is further compounded by talk of developmental programs, which Maynard-Smith raised when presenting a list of what biologists were justified in claiming for DNA:

DNA contains information that has been programmed by natural selection; that this information codes for the amino acid sequence of proteins; that, in a much less well understood sense, the DNA and proteins carry instructions, or a program, for the development of the organism; that natural selection of organisms alters the information in the genome; and finally, that genomic information is ‘meaningful’ in that it generates an organism able to survive in the environment in which selection has acted. (Maynard Smith 2000: 190)

In the diagram that accompanies these comments, Maynard Smith placed development directly after protein folding and labelled it as “‘channel conditions’ laws of physics + local environment” (p.191). By this he meant that whilst the laws of physics do not change, local environments do, and this introduces noise but not information to the process. He pointed out that genomes can evolve to allow plasticity and accommodation to such noise, and in so doing provide developmental robustness in the face of such perturbations. This was a part of his broader claim that research “in developmental biology is concerned with identifying regulatory genes, and with identifying the higher-level rules whose parameters the genes control” (p.192). This view is like that expressed by Mayr who also recruited information concepts.

The functional biologist deals with all aspects of the decoding of programmed information contained in the DNA code of the fertilized zygote. The evolutionary biologist, on the other hand, is interested in the history of these codes of information and in the laws that control the changes of these codes from generation to generation. (Mayr 1961: 1502)

Mayr claimed that the DNA code in the zygote controls the development of the individual, and he labelled DNA code “the program for the behaviour computer” of an individual (p.1504). For Mayr behaviour programs enabled plasticity and robustness solutions. But he was clear that this view of DNA code, and its role in development, did not allow for causal determination and precise prediction. Instead, he made a claim for indeterminacy in biology due to complexity, randomness, the uniqueness of biological entities and emergence. DNA may be decoded during protein synthesis, but the outputs of that process enter a complex system that reacts to various contingencies across ontogeny. What stability there is, is due to DNA coding preserved over time, what variation one sees in phenotypic outcome is in part a result of the higher-level rules and parameters that Maynard Smith referenced^5^.

### The Developmental View

The phenotypic gambit deployed within optimality models is, as said, an idealization. Complex causality is reduced to something more manageable to aid understanding. The concept of a developmental program is to some extent a corollary of this approach in that any introduction of developmental detail would not help in understanding adaptations as optimal solutions to fitness problems. However, in recent years some scholars have argued that this view has prevented the inclusion of developmental biology which has in turn prevented evolutionary theory from considering the emergence of form. Quite specifically, Pigliucci has called for the incorporation of a mechanistic theory of form into evolutionary theory as a part of the movement to extend the evolutionary synthesis (Pigliucci 2007). From this perspective, the emergence of the genes’ eye view from the latter stages of the Modern Synthesis period helped to exclude developmental biology, and to reduce the scope of evolutionary explanations (Uller et al. 2020).

One example of the developmental challenge to the Modern Synthesis has focused on the concept of reaction norms. Traditionally reaction norms capture the available phenotypic expression for the underlying genotype across a range of environments, and in this way suggest genetic control but also plasticity. During the Modern Synthesis views about reaction norms changed.

Wright saw this kind of plasticity as uncoupling the phenotype from the genotype to enable adaptive fit without the need for new genetic variation, while Schmalhausen regarded the norm of reaction as historically stabilized plasticity that enabled some fit with different environments. Dobzhanksy argued that it was the norm of reaction of the organism to the environment that changed during evolution, in other words, it was the plastic phenotype that was the focus of selection, and this brought gene frequency changes. In doing this, Dobzhansky brought the reaction norm to the population level such that the study of the adaptive norm was the study of all the genotypes, rather than of their phenotypes. (Dickins 2021: 149)

Sultan has recently criticized the Dobzhansky view of reaction norms, claiming that it ceded total control of the phenotype to the genotype (Sultan 2019)^6^, ignoring other developmental processes. Sultan reached her views under the assumption that the Modern Synthesis provided an informational view of genes as total instructions; a view that others have reached in light of the comments from Mayr and Maynard Smith (e.g. (Oyama 2000; Newman 2010, 2017)). Oyama has been particularly clear in her complaints about gene-centrism in evolutionary biology, claiming that the informational, or instructional view of the gene is straightforwardly preformationist, and she has argued for holism with regard to biological cause of form (Godfrey-Smith 2001). Oyama views development from a systems perspective where all resources have causal parity with respect to phenotypic outcome.

Evolutionary developmental biology (evo-devo) has provided criticism of gene-centrism within the context of discussion about the causes of form. Gene-centrism is seen as privileging the gene in causal accounts of development, a view not unrelated to that from Oyama. A key example of an evo-devo approach is the theory of facilitated variation. Kirschner and Gerhart see development as differentiation over time, delivered by hierarchically organized core developmental modules that can be differently regulated to produce phenotypic variation, requiring minimal extra genetic variation to create a novel specialization (Kirschner and Gerhart 2010). The developmental work within these modules relies upon dynamical processes, such as those seen when neurons seek muscle tissue by exploring space and dying off if they do not find any, or when dynamic microtubules are stabilized by a polarizing signal, to borrow their examples. This is a kind of selection process which does not rely on any instructional package but rather upon the physical properties of the system. This introduces a great deal of diversity, much of which is discarded in specific contexts. In other words, this diversity can only become useful when a new constraint, or system of constraints, emerge and cause novel stabilization.

Kirschner and Gerhart discuss dynamic systems that equivocate between at least two stable states and can be tipped into one or other with minimal input, a relationship described as weak linkage. These switch-like systems can be linked together, yielding great flexibility and responsiveness. This knife-edge stabilization is to be contrasted with systems where a specific value input (datum) would have a unique relationship with a context. This would be a case of strong linkage. Kirschner and Gerhart think that biology is predominantly characterized by hierarchically organized weak-linkage systems as they are more readily established and more flexibly enjoined. Strong linkage is, by definition, less flexible.

Following from these claims, Kirschner and Gerhart argue for a form of modularity, or compartmentation, that makes use of weak linkage. Each “spatial compartment in an embryo is defined by a small set of unique selector genes, which encode transcription factors or signalling molecules that are expressed uniquely in that compartment. The selector gene can then ‘select’ any other gene to be expressed or repressed in its compartment” (Kirschner and Gerhart 2010: 267). This order of genetic control is ongoing in all modules which leads to differentiation of form across the emerging organism as a set of parallel processes that can operate at different rates.

Kirschner and Gerhart show how this model of development can illuminate the three separate vertebrate inventions of the wing in pterosaurs, birds, and bats. Each involved a different compartmental modification leading to a similar emergent form that had necessary aerodynamic properties. The evolution of vertebrate wings relied upon conserved modular processes coming under novel regulation during development. Kirschner and Gerhart see that novel regulation as tipping dynamical systems within compartments into one state rather than another. They are claiming a core homology of basic developmental structure, that needed only a little change in control to give the phenotype novel direction and in each case those changes converged on a similar solution. One advantage of this organization is that where mutations within core modules are likely to be deleterious and disrupt essential building blocks for an organism, changes to regulation of those compartments will be less likely to be lethal giving great opportunity for the emergence of novelty. In this way phenotypic variation can be maximized relative to genetic variation.

Newman has proposed a similar developmental scheme beginning at the cellular level that relies upon the coordination of dynamical patterning modules (Newman 2010; Benítez et al. 2018). Newman first describes the cellular level:

The biosynthetic states of all cells are determined by the dynamics of transcription factor-mediated gene regulatory networks (GRNs)… Such networks, containing feedback and feed-forward loops by which the transcription factors promote and suppress their own and each other’s synthesis, exhibit multistability… The systems can thus switch among discrete states, the number of states always being much smaller than the total number of genes in the organism’s genome. Since the genes that specify nontranscription factor proteins and regulatory RNAs are themselves subject to transcriptional control, the alternative stable states of the GRNs specify cell types distinguished by extensive biosynthetic differences. (Newman 2010: 281-2)

The claim is that complex regulatory networks are going to produce multi-stable behaviour in cells because of their inherent physical properties, and stability caused in this way is not adaptation in the evolutionary sense. Newman (2010) argues that evolutionary processes reduce the tendency of networks to flip between states, by suppressing forms that might disrupt the emerging organism. Dynamic patterning modules in turn allow the emergence of spatial and temporal sequencing through ontogeny and the ordering of cell types produced by these networks (Newman 2010, 2017). These modules are “associations of specific gene products” and the “physical effects they are capable of mobilizing in the context of cell aggregates” (2017: 192).

This leads Newman to a claim that the gene-centric view is in error because genes do not carry information but instead act to specify building materials that then conform to the laws of physics – genes are not instructions for organismic design but merely antecedent causes of the material substrate. There is genetic control in the sense that gene products are specified, but then higher-level rules are embodied in physics.

### Summary

Where the genes’ eye view has permitted a form of optimality modelling, to better understand adaptation and evolutionary dynamics within populations, it has also relied on an idealization that reduces genes to instructions for phenotypes (Uller et al. 2020). This simplification removes developmental processes. This removal was made stark by discussion from Mayr and Maynard-Smith, in which they mooted developmental programs under the control of genes. In doing this they made explicit developmental comment, deploying information concepts, albeit within the context of optimality considerations. This has been seen as an error by those biologists and philosophers wishing to account for the emergence of form. Their claim is that gene-centrism has permitted an instructional view of the gene, missing many sources of phenotypic variation caused by developmental process that they claim are important for evolutionary dynamics.

## Biological Information

In this section I begin by outlining Maynard Smith’s (2000) position on biological information (3.1). His view was derived, in part, from Dretske’s (1983) work and so I continue with a brief discussion of this and related views on natural information (3.2). Following this I adopt and adapt Floridi’s (2010) general definition of information to claim that information should be seen as a functional relationship between inputs and their context (3.3). To this end the design of the context is crucially important, a view inherent in Maynard Smith and Mayr’s discussions (2.1), and one that does not conform to the instructional gene concept.

### Maynard Smith on Information in Biology

At the end of a natural history of uses of information in biology, Maynard Smith makes the following comment:

In colloquial speech, the word ‘information’ is used in two different contexts. It may be used without semantic implications; for example, we may say that the form of a cloud provides information about whether it will rain. In such cases, no one would think that the cloud had the shape it did because it provided information. In contrast, a weather forecast contains information about whether it will rain, and it has the form it does because it conveys that information. The difference can be expressed by saying that the forecast has intentionality…, whereas the cloud does not. The notion of information as it is used in biology is of the former kind; it implies intentionality. It is for this reason that we speak of genes carrying information during development, and of environmental fluctuations not doing so. ((Maynard Smith 2000: 192–193).

Maynard Smith’s claim is that the use of information concepts in molecular biology is colloquial, by which he means analogical. He makes clear that analogies work through formal isomorphism or qualitative similarity between the source and target (Shelley 2002; Frigg 2022), with the latter being more common. As examples of analogical uses of information, he lists *codes, redundancy, transcription* and *translation, messengers, proofreading, editing* and *libraries*. These concepts are strongly affiliated with a communicative view of information, derived from Shannon’s quantitative work (Shannon 1948). Thus, a communicative view of information is taken as a source to explain a target. From Maynard Smith’s list we can see that the target is broadly that of the role of DNA in protein synthesis.

Information in biology is often discussed with reference to Shannon’s work to improve the fidelity of transmission between a source and a receiver, via a communication channel (Shannon 1948; (Godfrey-Smith 2007b). This is seen as a weak sense of information because it is about correlation or contingent relation (Kumar 2014). A strong sense of information typically invokes meaning or content.

Shannon’s insight was to understand that the components of a signal could be arranged in several ways. For example, if one wished to convey the message *<A B C>*, it is possible to re-order this as *<A C B>*, *<B A C>* etc. This re-ordering has a limited range of six versions only one of which is the *true* message. The basic task of communication via a channel is to send a signal such that it arrives in the same compositional state that it was sent and so Shannon needed to develop a measure of the degree of conformity, how close to being *just-so* the received signal was (Cohen 2000). As Cohen notes, that *just-so-ness* of the message should stand out against the background possibilities. The more possibilities there are then the more surprising^7^ the arrival of the actual signal, and to this end probabilities can be attached to the likelihood of receiving a signal that is *just-so*. Shannon did not consider his theory of communication to be a theory of information, but rather one about the transmission of information (Deacon 2017). Information was assumed and not formalized by Shannon.

Maynard Smith notes that not everyone sees Shannon’s transmission of information model as a useful analogy. He addresses this within an evolutionary framework.

In the human example, a message is first coded, and then decoded. In the genetic case, although we think of a message in coded form in the mRNA being translated at the ribosome into the amino acid sequence of a protein, it is perhaps odd to think of this ‘de’-coding, since it was not ‘coded’ from protein to mRNA in the first place. I don’t think this destroys the analogy between the genetic case and the second part of the human sequence. But it does raise a hard question. If there is ‘information’ in DNA, copied to RNA, how did it get there? (Maynard Smith 2000: 179)

His answer is that natural selection puts the information into DNA. Natural selection, invoked as a process of design, has provided information that is passed from the DNA, via a communication channel consisting of ribosomes and mRNA, leading to a protein which can be said to have meaning understood functionally in fitness terms. Natural selection, regarded as a designer, lends this information intentionality in the sense invoked by Maynard Smith in the first quotation in this section.

### Natural Information

Maynard Smith’s position on information emerged from Dretske (Dretske 1983) in that it was a causal model where the protein outcome is caused by the DNA input (Kumar 2014). For Dretske, signals (smoke) carry meaningful information about their source (fire), because of their causal relationship. Meaning is something naturally occurring, and not in need of an interpreter but interpreters can make use of it. This relates to Shannon in that the correlation between smoke and fire is achieved via a channel, which we can assume is some kind of physical medium; there is a source of the relation; and, any receiver that is able to make use of the correlation has access to that meaning (Mann 2020). Crucially, Dretske is claiming that the relationship between smoke and fire does not depend upon any use of it. Information is understood in terms of raw causality, and that raw causality can later be adopted by agents who can make use of it. But Dretske sees that raw causality as capturing natural information (Kraemer 2015)^8^.

A common objection to Dretske has been that his model relies upon strong causality between such events as smoke and fire, and this is at best rare. This is often referred to as the reference class problem. Simply put, this means that not all smoke is indicative of fire, smoke can occur in the absence of fire. There is in fact a probable relation between smoke and fire, one which is < 1.0, and this suggests that smoke might not convey determined natural information: not least because there is no method for statistically determining what information smoke is conveying at a given point (Kraemer 2015; Mann 2020).

One solution to the reference class problem is to bring function into the relationship, in other words, to make use of it central. Maynard Smith has taken this next step and grounded information in natural selection as a source of the causal relationship between DNA and functional protein. Specifically, he invokes a second analogy of natural selection as a designer altering DNA sequences and delivering different causal outcomes with better fitness coefficients. This permits a form of *as if* intentionality – thinking about selection as an agent (Okasha 2018). This is apparently purposive work – in keeping with Mayr’s comments on teleonomic explanation in evolutionary theory (Mayr 1961) – and for Maynard Smith this justifies the idea that DNA contains information to be transmitted across generations, shifting from a weak to a strong sense of information as it is grounded in a view of biological intentionality or meaning (Kumar 2014). In the context of the reference class problem this solution provides a process that guarantees strong causality between DNA source and a particular protein outcome.

Maynard Smith gets a little tied up trying to justify the analogy in the second passage of his quoted above. His principal sticking point is the idea that the final message, the protein structure, was not the source that was encoded. Yet, he implies, biologists commonly talk of mRNA encoding protein structure^9^. In Dretskean terms we might simply talk of DNA sequence causing (strongly correlating with) protein structure, then refer to mRNA encoding DNA sequence, and leave it at that. Maynard Smith’s worry is based more in Shannon-type concerns relating directly to the mathematical theory of communication which was designed to solve an engineering problem that explicitly relied upon encoding and decoding. This perspective can be salvaged if we understand mRNA as encoding DNA sequence as a complement to allow transmission from the nucleus. What happens next at the ribosome is decoding via tRNA and the formation of a polypeptide chain and a protein structure. This latter set of actions could instead be seen as a use of the decoded sequence further downstream. Put more simply, the Shannon-like process is confined to a DNA→mRNA→tRNA transmission scheme, where fidelity can be quantified using Shannon’s mathematical model (Yockey 2005).

Some efforts have been made to locate meaning in the downstream uses of the decoded DNA sequence. As Kumar (2014) discusses, a biosemiotic Peircean view that relies upon an interpreter to make sense of a produced sign has been applied to protein synthesis. The interpreter of the DNA sign might be the entire distributed protein-synthesis machine and the meaning of the DNA code is produced by this interpreter (Godfrey-Smith 1999)^10^. This is a teleosemantic view and is most associated with Millikan.

(A) good look at the consumer part of the system ought to be all that is needed to determine not only representational status but representational content. We argue this as follows. First, the part of the system which consumes representations must understand the representations proffered to it. Suppose, for example, that there were abundant “natural information” (in Dretske’s sense) contained in numerous natural signs all present in a certain state of a system. This information could still not serve the system as information, unless the signs were understood by the system, and, furthermore, understood as bearers of whatever specific information they, in fact, do bear… So there must be something about the consumer that constitutes its taking the signs to indicate, say, *p, q*, and *r* rather than *s, t*, and *u*. But, if we know what constitutes the consumer’s taking a sign to indicate *p*, what *q*, what *r*, etc., then, granted that the consumer’s takings are in some way systematically derived from the structures of the signs so taken, we can construct a semantics for the consumer’s language. Anything the signs may indicate qua natural signs or natural information carriers then drops out as entirely irrelevant; the representation-producing side of the system had better pay undivided attention to the language of its consumer. The sign producer’s function will be to produce signs that are true *as the consumer reads the language*. (Millikan 1989: 286).

Thus, the protein synthesis machinery can be said to understand the proffered tRNA code at the ribosome. This understanding is nothing more than a systemic readiness to change states upon receipt of certain inputs. The machinery is designed (by evolutionary process) to respond in a delimited set of ways to such inputs. That delimitation affects probabilities and appears to permit a form of Dretskean natural information because of correlation between DNA and protein. This is a pragmatic view of natural information, where its meaning might be seen as dependent upon the contexts in which probabilistic correlations are derived.

### Natural Information and Systemic Context

Floridi has made the case for Shannon developing a theory of *data* (Floridi 2010). Under this interpretation information is a property of a system that can be in more than one state. The precise state that a system is in is determined by an input. If a system can exist in *S*_*1*_ to *S*_*n*_ states, an input which causes it to change from *S*_*1*_ to *S*_*2*_ is *informative*^11^, but the input is not information. Instead, the information resides in the new state of the system, understood as a function of the input. Floridi clarifies this using the example of a dedicated computer awaiting the outcome of a fair coin toss. The computer is in a state of *data-deficit* prior to tossing the coin, which Shannon referred to as a state of *uncertainty*: the system can be in *n* states, but a precise state, *S*, has yet to be determined. This will be determined by inputting the outcome of the toss, and that input is a *datum*.

In this example, tossing the coin yields an amount of information as a function of two equiprobable outcomes, *<heads>* or *<tails>*. Using Shannon’s quantification this is 1 bit of information calculated as *log*_*2*_*2 = 1* and is equal to the data deficit it removes. This can be understood as a measure of uncertainty if one considers the number of *yes* or *no* questions needed to determine which side up the coin had landed after a toss: *<Is it tails?> <Yes.>*.

As Floridi notes, the idea that information can be *quantified* in terms of the reduction of uncertainty does not tell us what information is. This becomes clear when we realize that one can produce two equal amounts of information about two entirely separate objects. For example, both *< can I have chips with that?>* and *< is it vegan?>* can be answered with a *< yes >* or *< no >* yielding 1 bit of Shannon-information. But this quantification does not help in understanding what role the Shannon-information has. Floridi clarifies this point using a general definition of information, such that:

$$Information = data + meaning$$

In using this formulation Floridi is apparently appealing to a strong sense of information (Kumar 2014) because data must relate in some way to the semantics of the system it enters to be considered informative. The relation, in this case, is one of conjunction. To this end, information is the functional outcome of a relationship between data and semantics. Floridi uses this formulation to then express the quantification of information as:

$$Information - meaning = data$$

This, he argues, demonstrates that Shannon’s mathematical theory of communication is in fact a theory of data communication.

My use of the query structure above was borrowed from Floridi. The query *< is it vegan?>* can have a binary response, but the query provides semantic context. That context allows for two possible states – the object is either vegan or not. If this query is answered with a *<yes>* that answer is to be regarded as a datum that then yields 1 bit of Shannon-information as it completes the informational relationship offered by the query.

Floridi’s position helps us to see that information is not to be reified, not to be seen as something that can be harvested, but rather as the functional outcome of a relationship between data and meaning. Boisot and Canals have clarified the distinction between data and information by reference to cryptography (Boisot and Canals 2004). One can access a dataset but if it has been encrypted then that data cannot be used, it cannot be inputted into relevant contexts. This tells us that the informational relationship is dependent upon the fit of data to a context. Floridi captures this idea of context by making ready reference to semantics, to the meaning of the query in the kinds of examples given above. But more mechanistically, we only require the input to change the state of the system it is entered into to deliver a minimally informational relationship. Given this I prefer to rephrase Floridi’s general definition as follows:

$$Information = data + context$$

This is again a conjunction. I am using the term < context > to denote a system that is prepared in some way to accept certain data. The system in this way provides a context for data of a certain kind, and the intuition is that the nature of that kind is something that results from the design of the context/system. Design can be the act of an agent or the result of a process like natural selection, which we can treat as agential (Okasha 2018). Data of the right kind for the context will change the state of that context. When that functional conjunction happens, we might say that the context, or the system, has been informed but this sense of information captures the whole interaction between data and context. Information is not data alone, nor is it context alone, it is a relationship. We can note it when we see state change^12^.

What are *data*? According to Boisot and Canals data “can be treated as originating in discernible differences in physical states-of-the-world” (2004: 46). Their view of *data as stimuli* is one based in energy such that there are energetic regularities in stimuli. They discuss this in neurological terms, noting that a neurological mechanism will fire, or switch when energetic thresholds are exceeded causing a shift in the equilibrium state of that mechanism. Mechanisms are to be regarded as those things that direct available (or free) energy to do work, and work amounts to systemic state change (Pattee 2001; Bechtel and Bich 2021; Dickins 2021b). It is implied that different mechanisms will respond to different stimuli, and thus to different energetic regularities. Boisot and Canals see agents as those things that discern stimuli, but their view of agents is wholly mechanistic and relies upon a commitment to a naturalistic theory of design, something provided by evolutionary theory. Evolutionary process takes advantage of traits that can usefully discern. Under this interpretation traits are to be seen as contexts that differentially make use of available energetic inputs. As a result we can, for example, sketch an evolutionary story of increasingly complex neurological agents, all based upon binary 1-bit neurons (firing or not) organized into networks that provide an ability to discern increasingly subtle energetic regularities and coordinated via ganglia to control specific systems such as motor outputs (Keijzer et al. 2013; Keijzer 2015). Organisms (or agents) are thus a mechanistic super-context comprised of a heterarchy of sub-contexts. Each context may be regarded as specialized to discern and respond to specific data, and in so doing an informational relationship emerges such that the context, the agent, the organism is informed about an external (or internal) contingency and adjusts its state accordingly^13^.

This is not unrelated to a recent proposal from Scarantino (2015):

The Probabilistic Difference Maker Theory (PDMT) of natural information that I offer here is defined by three core ideas: (i) natural information must be captured by a three-place relation between signals, states of affairs, and background data, (ii) carrying natural information about a state of affairs simply amounts to changing its probability relative to background data, and (iii) natural information can be quantified using a measure inspired by Shannon’s communication theory and Bayesian confirmation theory. ((Scarantino 2015) pp. 419–420)

This theory is addressed to the processing of signals in organisms of varying levels of complexity. The idea is that signals will naturally relate to their sources, which are states of affairs in the world. But for a signal to carry natural information about the source, the organism must also have background data about the probability of the signal being caused by the source. So, smoke can be treated as a signal of fire in the context of prior probabilities about the causal relationship between fire and smoke. Signals can variously confirm or disconfirm these priors and Scarantino packages this in an explicitly Bayesian manner. This approach removes the obstacle of the reference class problem for a more Dretskean account, and effectively places background data in the consumer or interpreter role developed by Millikan. To this end the presence of smoke significantly increases the probability of a fire being nearby for most consumers of this signal with relevant background data^14^. In Floridian terms, this theory can be seen as demonstrating how contexts can be increasingly sensitized by data, changing the probability of particular state changes in the presence of specific data tokens over time.

A strength of Scarantino’s proposal is that it does not rely on a nomic (lawlike) relation between source and signal but can also allow for learning, where an agent populates its background data through gradual exposures (cf. (Skyrms 2010; Wagner and Franke 2013)). With each new signal-source pairing priors are updated, as we see in classical conditioning or expectancy learning. In this sense there is a generality to the proposal, but we can also see that the focus is at a psychological or cognitive level of biological organization. Nonetheless, the concept of a data sensitive context, understood as a system that can change states, fits Scarantino’s broad view of data. As with learning, where natural selection builds contexts, it is embodying data about historical conditions – this is the nature of selection.

### The Limits of the Information Analogy

What I have just sketched describes the scientific agenda of Maynard Smith (2000). Maynard Smith sought to use communication as an analogy for protein synthesis, but in doing this he was not committing to DNA acting as a complete instruction for final form. Indeed, his concerns about the analogy breaking down (3.2) were a realization of the limits of the analogy. The coding and decoding of DNA sequence during protein synthesis would not account for the totality of protein form and function, as that end point was not transmitted. Instead, this relied on protein folding and “‘channel conditions’ laws of physics + local environment” (Maynard Smith 2000: 191). What DNA provided was a transmitted data set that acted to change the state of the protein synthesis machinery. This is also why Shannon did not regard his theory of communication as a theory of information – other processes were involved to turn data informative. The teleosemantic view of this machinery as an interpreter is best understood as a view of data sensitive contexts that can systematically change state, and in ways that impact upon fitness.

## Back to Development

In this section I will revisit the developmental issues covered in Sect. 2 and apply the view of information developed above. First, I will discuss protein folding, which Maynard Smith raised (4.1) and then reaction norms (4.2) and developmental systems (4.3). My ambition is not to present a synthetic view of developmental processes in this section, but merely to bring prior examples back into focus using the perspective endorsed in this paper.

### Protein Folding

When the claim is made that DNA should be understood as data inputted to a context, those contexts are understood mechanistically as systems that can change state in regular ways in response to that input. For example, during protein synthesis, the DNA→mRNA→tRNA transmission scheme can be unpacked as data effects. DNA *causes* a complementary mRNA. mRNA is then inputted to ribosomal mechanisms which *cause* the arrival of a particular sequence of tRNA, each carrying an amino acid. This in turn *causes* the formation of a polypeptide chain of amino acids that are then folded into a protein.

Protein folding has been a significant empirical and theoretical challenge for biology since the discovery of DNA structure (Powers and Gierasch 2021). It is known that the sequence of amino acids in a polypeptide chain, which is caused by DNA sequence, determines the three-dimensional folded structure of a functional protein. In recent years our ability to predict structure from sequence has been greatly enhanced by machine learning techniques (AlQuraishi 2019; Jafari and Javidi 2020; Noé et al. 2020). We might claim that these processes improve the probabilities of sequence→protein structure predictions by deploying an agent (the machine learning algorithm) to explore design space, in this way pursuing a deep causal relationship (Scarantino 2015).

For the purposes of this paper, it is interesting that DNA sequence determines amino acid sequence that in turn can be seen to determine three-dimensional protein structure, and it is this structure that confers a function to a protein. This is the core of the information flow approach to biology, as we have seen. But what is also known is that the sequence of amino acids on a polypeptide chain enables different amino acids to interact locally, and it is those interactions that cause folding and effectively reduce the search space of possible configurations for the emerging protein (Zwanzig et al. 1992). Folding reduces available (free) energy to a minimum in a stable, native (natural) protein, but other stable minimum free energy configurations are possible, some presenting as mis-folded. These mis-folded proteins can be extremely deleterious to the organism, leading to a number of diseases in humans, e.g., Alzheimer’s and Parkinson’s diseases (Reynaud 2010). As Powers and Gierasch note, there are mechanisms to marshal protein folding away from non-native but nonetheless stable configurations. Under a strict sequence to folding prediction, non-native proteins would be regarded as incorrect solutions but in fact they should be seen as localized thermodynamic possibilities that require extra process to avoid, and it is known that a number of co-factors, including enzymes classed as foldases, provide this service. Such extra process should be favoured by selection and there is evidence to suggest that co-factors have been an essential part of the evolution of functional proteins since the beginning of life (Chu and Zhang 2020)^15^. The fact of these extra processes, and an increasing awareness of cellular environmental effects, leads Powers and Gierasch to conclude that many of the causes of three-dimensional protein structure are extrinsic to the sequences. In this way sequence is a necessary but not sufficient antecedent condition for native protein formation during development.

Given the above account we can talk of protein folding as a kind of mechanism where free energy is diverted to do work that leads to state change and the minimization of free energy upon completion (Bechtel and Bich 2021). Furthermore, we can see that specific DNA data inputs lead to specific states in protein space through the causal pathway just outlined. The rules of protein folding are determined by physical constraints imposed from within the amino acid sequence and from without by co-factors. This is a step further along a broadly construed developmental pathway and not fully a property of the DNA code that caused the polypeptide sequence. It is nonetheless in accord with the idea of DNA as data that enables state change within the context of ribosomal machinery and amino acids. And it is in accord with the idea of higher-order rules of development. Maynard Smith’s (2000) notion that these rules are under genetic control appears justified in the case of protein folding. The genetic control is delivered by providing amino acids with specific amino acid neighbours, facilitating local folding. But the control is not total. The idea that genes are necessary but not sufficient resources for this aspect of development is implied by the requirement for higher-order rules (Maynard Smith 2000).

### Reaction Norms

Under the view of information discussed in this paper, reaction norms should be seen as the product of systems designed to respond to an array of environmental inputs. Those inputs are data, and the development systems are responding systematically to it. As such there is information in the sense of a functional relationship between environmental input and organism, the organism is developmentally responsive. Those developmental systems are constructed with genetic input, and that input is a necessary but not sufficient cause of the system. The environmental inputs are also necessary input, with respect to final form. We can nonetheless model the selection of reaction norms in optimality terms and continue to take the phenotypic gambit when our question is about fitness returns and stable strategies at the population level. This idealization necessarily misses the detail of the developmental process, and the long history of how plastic phenotypes influence selection, because it is simply irrelevant to the task. Uller and colleagues (Uller et al. 2020) have recently claimed that this idealization, in preventing a focus on that long history, somehow reduces the richness of evolutionary biology, and they present examples of such accounts to demonstrate how different they are. But this does not amount to a criticism of the gene’s eye view, merely a restatement of its purpose. From the outset the gene’s eye view was designed to simply to tell a specific story – or a specific story simply. That it does not also tell a different story is of no relevance.

### Developmental Systems

In an early paper on developmental systems, Griffiths and Gray discussed two ways to incorporate information into developmental biology (Griffiths and Gray 1994). The first was to see all developmental resources as information, which is close to what Oyama advocated (2.2). The second, which they see as easier to implement, is to pragmatically embed information in just one resource and regard all others as channel conditions. They carefully note that there is no principled reason for choosing one resource over the other as the informational source in this second case.

I think Griffiths and Gray have correctly presented two available options for information concepts within developmental biology. And I think that those criticizing gene-centrism see Mayr and Maynard Smith as having opted for the second choice, labelling DNA as the source of (intentional) information. It is the case that DNA was explicitly labelled as information by both theorists. But stating this sidesteps the reasons for that choice. Neither theorist was attempting to comment on development, but rather upon evolution. Both firmly saw evolution in terms of changes in gene frequencies and both saw DNA as holding information (in their terms) constant across generations. This idea is at the heart of the replicator-vehicle concept: vehicles reproduce inaccurately, replicators copy faithfully. But, both Mayr and Maynard Smith saw DNA as inputs to higher-level systems, or into other contexts, where effects are wrought, and development occurs. In this way they were really discussing data. When thinking as developmentalists there is nothing in their work that deviates from the parity considerations of Oyama and other developmental systems theorists, and this further reinforces the view that the information talk during the Modern Synthesis was colloquial but served as an idealization to capture the causality of data in multiple contexts^16^.

Returning to the modular and regulatory approaches from evo-devo (Kirschner and Gerhart 2010; Newman 2010) we can see that this work conforms to the higher-order rules idea of Maynard Smith. Genetic inputs are essential to change states within compartmentalized developmental units, and the downstream state changes rely, as with protein folding, on non-genetic causes. Again, as with protein folding, this makes genetic input necessary but not sufficient as a cause of form. But, when it comes to modelling stable forms in optimality terms, the idealizations of the phenotypic gambit remain appropriate precisely because of this necessary relationship between genes and phenotype.

While complex life relies upon non-genetic physical processes and properties for its formation the view of information outlined in this paper demands a counterfactual check when thinking about development. Simply put, can we imagine development without genetic inputs? The answer will be no, under current scientific models.

## Summary and Conclusion

Drawing together the threads of the paper, the following argument can be made:

The instructional interpretation of genetic information, associated with the Modern Synthesis, is an idealization when applied to development. As an idealization it pushes all the causality into the gene because the Modern Synthesis was focused upon evolution as changes in gene frequency. The Modern Synthesis only needed to assert a role for genes in development, to allow each new generation to emerge, and to be approximately like the previous, selection permitting. Just so long as development happened in this way, evolution could continue.

Despite this idealization key theorists of the Modern Synthesis did enough housekeeping to make sure that the concept of information in play broadly captured developmental parameters. In this paper I have discussed Mayr and Maynard Smith in this capacity, but even Dawkins discussed the catalytic role of genes in development (Dawkins 1976). They were clear that genes did not in fact determine all aspects of emergent form, and that there was a behaviour program or set of higher-level rules that responded to genetic input. When making these points they were much closer to the view of information as a functional relationship between data and context, between input and system. This is most apparent when Maynard Smith argued for intentionality bequeathed by natural selection to ground a biological concept of Dretskean meaning.

When inspecting accounts of protein folding, reaction norms and evo devo, all the models used conform to the idea of information as data + context. Genetic inputs delimit physical processes, leading to a greater propensity to stability of form. In this way genes are data affecting state change. Reaction norms are to be seen as high-level responses to environmental inputs. The mechanics of such responses are mediated by morphological, physiological, and behavioural systems all of which will have developmental trajectories that may well operate according to the broadly modular principles outlined by evo-devo. Reaction norms simply describe what a developing organism can respond to, the limit of the higher-level rules that genes act to control but not fully determine.

So, we are left with a view that the use of genetic information concepts within evolutionary accounts relied upon an idealization of the role of genes in development to focus on evolution. That idealization was developmental, in that the gloss position could be expressed as *evolution requires genes, and those genes directly instruct development*. The untruth here was in the second clause. However, the housekeeping that Mayr and Maynard Smith undertook was about that second clause. Maynard Smith took time to extend the use of information into a discussion about where development was positioned and the role of genes in controlling higher-level developmental processes. In doing this he admitted the complexity of development and posed a role for genes in that complexity. In this way his informational account, especially when reinterpreted in light of Floridi’s view on data, becomes an abstract representation of development with much omitted detail but no specific untruths (Levy 2018). We can make this latter claim stick because of the way in which protein folding, reaction norms and evo-devo place genes in their accounts. For example, the modular organization of facilitated variation or dynamic patterning programs simply layers on detail to the abstractions of Maynard Smith.

What is interesting is that Maynard Smith, and doubtless many others within the discipline of evolutionary biology, moved seamlessly between discussions of genes as information in evolution and in development. When this is done in the company of developmental biologists it is easy to see how they might lose track of what is in fact being claimed. To this end the lesson to take from the developmental challenge to the Modern Synthesis might be that more precision is needed when articulating just what information is. In the view espoused here it is not a thing, but a functional relationship. But once that is articulated it is plain to see that the Modern Synthesis deployed a version of this position to great effect and that the challenges from development do nothing to that explanatory framework.

## Footnotes

1 Throughout the paper I am assuming that the characterization of the Modern Synthesis as a paradigm, rather than an extended historical period of theoretical and empirical development leading to a plurality of concerns and approaches, is valid. As this footnote suggests not all historians agree with this view (Smocovitis 1996; Delisle 2017).

2 There are many available gene concepts – this paper deals with just one. The arguments I present may or may not apply to some of the other concepts available (cf., Falk 1986, 2010; Keller 2000). It should also be born in mind that the period of the Modern Synthesis began with a non-DNA concept of the gene (Johannsen 1911), and undoubtedly vestiges of those pre-DNA concepts affected biological theory beyond the introduction of DNA concepts (Mayr 1982; Smocovitis 1996; Provine 2001; Bromham 2016; (Dickins 2021a).

3 Hull derived a similar scheme (Hull 1980).

4 This was also a simplifying assumption, such that survival at the expense of another might improve future fitness by increasing food share and enhancing development, making any ejector more competitive in other arenas.

5 Readers may be aware of Mayr’s critical cartoon of population genetics as beanbag genetics. This was a catchphrase to capture his discontent with simplistic population genetic models that focused on gene frequencies and not the operation of gene complexes, and other processes, to produce the phenotype. His criticism did not amount to a denial of the role of genes in evolution, but rather to a plea for greater sophistication in modelling (Rao and Nanjundiah 2011).

6 The foundations of Sultan’s criticisms are to be found further back in time, when she takes explicit aim at the Modern Synthesis and gene-centrism in the context of discussion about plasticity (Sultan 1992).

7 This property is referred to as *surprisal* and is the measure of the probability of a particular input occurring against the backdrop of a particular context. This is related to an entropy concept, which is the expected amount information to be returned.

8 There is a sense in which Dretskean natural information is equivalent to the concept of affordance from ecological psychology following Gibson (Gibson 1979). Natural information is structured patterning in the external world that organisms can discover and use, and for Dretkse the meaning is packaged within the correlation and is not dependent upon internal states of the organism. Mann notes Dretske did concede that some information was only accessible if the organism had sufficient background knowledge (Mann 2020). But background knowledge could be packaged pragmatically, and in evolutionary design terms, to save this ecological point.

9 mRNA, or messenger RNA, is a single stranded complementary copy of one strand of DNA. It passes from the nucleus of the cell into the cytoplasm where it meets with a ribosome. Ribosomes effectively read the mRNA strand one codon (three bases) at a time, and this enables complementary tRNA (transfer RNA) to join with it. Each tRNA is associated with one amino acid. Those amino acids form a chain in a sequence that is determined by the codon sequence originally sourced in the DNA strand. That chain then folds into a protein, a process that has recently received new insights (AlQuraishi 2019).

10 Godfrey Smith actually relies upon Millikan’s distinctions between producers and consumers, but as Kumar points out these distinctions relate directly to Peircean semiotics (Millikan 1989; Kumar 2014).

11 Cohen (2000) notes the etymological relationship between *form* and *information*, the idea of changes in arrangement.

12 My intuition is that the semantics Floridi relies upon for his examples are in fact gloss descriptions of *data + context* relationships within human cognitive systems. So, a concept such as [vegan] is really a network property of the brain. In other words, it is possible to see semantics as mechanistic context all the way through ((Dickins 2021c). But this is not essential to my argument and those who like to treat semantic content as a special form of context can continue to do so for these cases.

13 Note that I am assuming the super-context delivers a unity of purpose such that agent-talk is both permitted and useful (Okasha 2018).

14 Haig has recently developed a closely related view in which an interpreter “is an evolved or designed mechanism that couples *possible* inputs… to *possible* outputs” (Haig 2020) p.310). The mechanism is uncertain until an actual coupling occurs, and for Haig informational inputs are difference makers, whilst meaning is to be seen as the actual difference made.

15 These enzymatic co-factors are themselves proteins thought to have emerged from early primitive peptide structures with catalytic functions. These antecedent conditions enabled the selection of the protein synthesis machinery we see today (Chu and Zhang 2020).

16 Griffiths and Gray also view information concepts as idealizations designed to capture the more complex causal interactions of inputs and mechanisms.
